# Organic Amendments Regulate Soil Bacterial Diversity and Cooperative Network Structure in Reclaimed Coal Gangue Soil

**DOI:** 10.3390/microorganisms14010017

**Published:** 2025-12-20

**Authors:** Zeyu Zeng, Tao Kong, Gang Lv, Haotian Cheng, Sinuo Bao, Lin Xiao

**Affiliations:** 1 College of Environmental Science and Engineering, Liaoning Technical University, Fuxin 123000, China; zzyxb98@163.com (Z.Z.); lvgang2637@126.com (G.L.); bsn08219819@163.com (S.B.); linx00787@gmail.com (L.X.); 2 Institute of Applied Ecology, Chinese Academy of Sciences, Shenyang 110016, China; chenghaotian@iae.ac.cn

**Keywords:** photovoltaic agroforestry system, residue after evaporation (RAE), cattle manure, *Trichoderma* bioinoculant, co-occurrence networks

## Abstract

Restoring soil microbial functioning in reclaimed coal gangue soils is critical for ecosystem recovery, yet how different organic amendments, particularly industrial by-products, regulate bacterial communities remains unclear. Here, we tested three organic inputs—the residue after evaporation (RAE) from vitamin C production, *Trichoderma* inoculation, and cattle manure—applied alone and in combination in a photovoltaic agroforestry system on coal gangue spoil. Our results indicate that the treatment based on manure increased bacterial α-diversity and favored taxa associated with organic matter transformation, including Actinobacteria and Acidobacteriota, suggesting expanded niche partitioning in response to heterogeneous substrates and nutrients. RAE alone supported communities closer to non-manure controls but, when co-applied with manure, further enhanced network connectivity and the prevalence of positive associations, indicating strengthened cooperative interactions and functional redundancy. In contrast, RAE combined with *Trichoderma* in the absence of manure reduced diversity, and simplified the co-occurrence network, suggesting resource monopolization and antagonism. Overall, RAE acted as a key driver of microbial cooperation and potential ecosystem resilience, and RAE-based amendments, particularly when integrated with manure, appear to be effective strategies for improving soil microbial functionality in degraded coal gangue soils.

## 1. Introduction

Mining operations have significantly disturbed terrestrial ecosystems, especially in arid and semi-arid areas, by compromising soil structure, diminishing nutrient availability, and hindering the recovery of vegetation [1,2]. Central to mitigating the impacts of mining is the rehabilitation of mined landscapes, with the ultimate goal of establishing aboveground flora and fauna that exhibit adequate composition and diversity [3,4]. In recent years, agrivoltaic systems—which integrate photovoltaic (PV) energy generation with the cultivation of vegetation—have surfaced as a novel means for land reclamation [5]. This strategy of dual land use enhances efficiency while also aiding in soil stabilization, regulation of microclimates, and carbon sequestration within degraded regions [6]. Nonetheless, the primary barriers to establishing vegetation beneath PV installations are poor soil fertility and restricted microbial activity [7].

To address these limitations, biological soil improvement strategies that integrate organic amendments with functional microorganisms have gained increasing attention [8]. Organic substrates, such as livestock manure, enhance soil fertility by providing diverse carbon and nutrient sources that stimulate microbial metabolism and enzyme activities, thereby promoting nutrient cycling and soil aggregation [9,10]. Meanwhile, industrial byproducts, such as the residue after evaporation (RAE) from vitamin C production, represent a promising source of organic matter for ecological restoration [11]. RAE is rich in low-molecular-weight organic acids and soluble carbohydrates, which can serve as labile carbon sources to activate soil microbial processes, increase enzymatic hydrolysis rates, and improve nutrient bioavailability [12]. Recent studies have shown that the controlled application of RAE improves soil physicochemical properties and promotes plant growth in degraded soils [11]. In parallel, *Trichoderma* species are well-known rhizosphere fungi that enhance plant growth, suppress pathogens, and promote nutrient transformation through the secretion of enzymes and bioactive metabolites [13,14]. When applied to nutrient-poor soils, *Trichoderma* can accelerate organic matter decomposition and increase microbial turnover, thereby enhancing soil biochemical functions and resilience [15,16]. Nevertheless, the combined effects of RAE and *Trichoderma* under agrivoltaic conditions remain largely unexplored [11,12]. Understanding how substrate quality and microbial inoculants interact to shape soil microbial communities is therefore critical for optimizing reclamation strategies in mining ecosystems [17].

Beyond improving soil nutrient status, organic amendments profoundly influence soil bacterial communities, which play pivotal roles in regulating nutrient cycling, organic matter decomposition, and ecosystem resilience [18,19]. Bacteria are the primary drivers of carbon and nitrogen transformations in soil, mediating enzymatic hydrolysis, mineralization, and the synthesis of humic substances [20]. Changes in substrate composition—such as the input of labile organic acids and carbohydrates from residues like RAE—can alter bacterial metabolic pathways and shift the balance between copiotrophic and oligotrophic taxa, thereby influencing soil biochemical stability [9]. Moreover, enhanced bacterial diversity and connectivity strengthen functional redundancy, enabling soils to better buffer against environmental disturbances [12,18]. Consequently, elucidating how organic amendments restructure bacterial community assembly and interaction networks is critical for understanding the mechanisms of soil fertility restoration in degraded ecosystems.

In this study, we established an agrivoltaic intercropping system in a mining region of western Liaoning, China, to investigate how RAE, *Trichoderma*, and their combination affect soil bacterial diversity, community composition, and co-occurrence network structure. Specifically, we aimed to (i) assess the effects of single and combined organic and microbial amendments on soil physicochemical and biochemical properties; (ii) elucidate how these treatments reshape bacterial community diversity and assembly patterns; and (iii) explore the mechanisms by which resource quality and microbial interactions jointly influence soil ecosystem functioning. We hypothesized that manure-derived substrate heterogeneity and the addition of RAE would expand microbial niche space and promote cooperative interactions within bacterial communities. The results provide new insights into the resource–microbe–network coupling mechanisms that underpin soil microbial reorganization and ecosystem recovery under agrivoltaic conditions in mining regions.

## 2. Materials and Methods

### 2.1. Study Site Description

**Figure 1:**
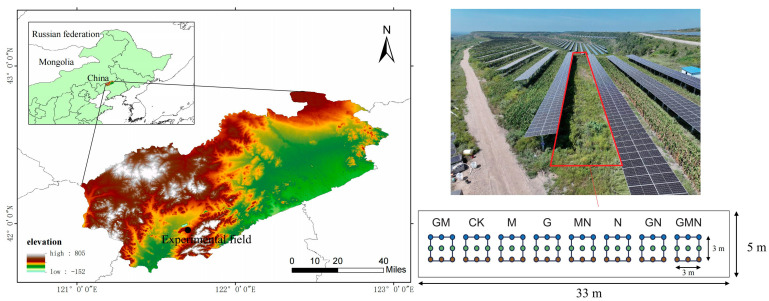
Information on the experimental site. The experimental plot was located within a photovoltaic agricultural system. The total plot length was 75 m, with photovoltaic panel columns spaced at 13 m intervals. A 33 m section at the center of the plot was selected, within which a 5 m wide open area free from photovoltaic shading was designated as the experimental plot. The nine points indicate nine planted sea buckthorn individuals, and soil samples collected according to color were pooled into one composite sample. CK, control; G, RAE; M, *Trichoderma*; N, cattle manure; MN, *Trichoderma* and cattle manure; GM, RAE and *Trichoderma*; GN, RAE and cattle manure; GMN, RAE and *Trichoderma* and cattle manure.

The research area is located at 121°42′21′′ E and 41°57′42′′ N, situated in the ecological restoration zone of coal gangue piles in the western part of Liaoning Province (Figure 1). The terrain in this region is generally undulating, with an elevation of approximately 200 to 250 m. The predominant landform types are eroded residual hills and gentle slopes. The area experiences a typical temperate continental monsoon climate, with an average annual temperature ranging from 7 to 9 °C and annual precipitation of approximately 450 to 550 mm, mainly concentrated between July and September. The potential evaporation exceeds 1500 mm, indicating a significant moisture deficit.

### 2.2. Experimental Design

The experiment adopted a randomized complete block design to evaluate the individual and interactive effects of the residue after evaporation (RAE) from vitamin C production, *Trichoderma* and well-decomposed cattle manure on the growth of sea buckthorn (*Hippophae rhamnoides* L.) and soil microbial regulation. Eight treatments were established: CK (control), G (RAE alone), M (*Trichoderma* alone), N (cattle manure alone), GM (RAE and *Trichoderma*), GN (RAE and cattle manure), MN (*Trichoderma* and cattle manure), and GMN (RAE and *Trichoderma* and cattle manure). The cattle manure used in this study was well-composted manure derived from Simmental cattle, whose diet consisted primarily of maize and wheat bran. In each treatment plot, nine uniformly growing sea buckthorn seedlings were planted and used as replicates, with the individual tree considered as the experimental unit. One-year-old sea buckthorn seedlings were pruned to 40 cm before planting, and transplanted on 4 May. Each treatment was assigned to a 3 m × 3 m plot, with seedlings planted at 1 m × 1 m spacing and 1 m buffer rows maintained between adjacent plots.

RAE was supplied by Northeast Pharmaceutical Group Co., Ltd. (Shenyang, China). The initial pH of RAE was approximately 1, making it unsuitable for direct use in plant cultivation. To minimize soil pH fluctuations, RAE was diluted 200-fold and adjusted to pH 6.3 using NH_3_·H_2_O under water-bath conditions prior to application, ensuring that the molecular structure of low-molecular-weight organic acids (LMWOAs) was preserved. After the seedlings had recovered from transplanting, RAE was applied regularly during the growing season from May to August at a rate of 40 mL per plant per application, with an interval of 10 days between applications, for a total of approximately 10 applications. The diluted RAE was applied as a basal drench around each plant to ensure infiltration into the root absorption zone. *Trichoderma* spp. was obtained from Guangzhou Weiyuan Biotechnology Co., Ltd. (Guangzhou, China). The product was a water-soluble powder containing 2 × 10^9^ viable cells g^−1^, which is readily activated upon hydration and applied to improve soil health. For the *Trichoderma* treatment, a commercial *Trichoderma* spp. formulation was applied at 4 g per plant directly to the root zone in the planting hole on the day of transplanting, followed by backfilling and thorough watering. For the cattle manure treatment, 1000 g per plant of fully decomposed cattle manure was applied in a ring around each plant on the day of transplanting. No additional chemical fertilizers were applied during the sea buckthorn growing period.

### 2.3. Soil Sampling

Soil sampling was conducted in October 2024. Before collecting soil cores from the 0–20 cm layer, surface litter and debris were removed from each experimental plot. For each treatment, three soil cores were taken from each row of sea buckthorn and thoroughly homogenized to obtain one composite soil sample per row. With three rows per treatment, a total of 24 soil samples were collected. After sampling, visible roots and stones were removed, and the soils were air-dried and passed through a 2 mm sieve. The processed samples were then kept refrigerated during transport to the laboratory and stored at −20 °C until analysis. One subsample was used for determining soil physicochemical properties, and another subsample was used for the assessment of microbial and biological indicators.

### 2.4. Soil Environment Factor

Soil pH was measured in a 1:2.5 (soil/water) suspension using a glass-electrode pH meter (PHS-3C, INESA, Shanghai, China). Soil organic carbon (SOC) was quantified using the K_2_Cr_2_O_7_–H_2_SO_4_ dichromate oxidation method. Soil available nitrogen (AN), phosphorus (AP), and potassium (AK) were determined using the alkaline hydrolysis diffusion method, Bray’s No. 1 method, and ammonium acetate extraction method, respectively [21]. Soil enzyme activities were assessed through established colorimetric techniques [22]. The measurement of urease activity (UE) in the soil was conducted using the sodium hypochlorite colorimetric approach [23]. Sucrase activity (SUC) in soil was evaluated via the 3,5-dinitrosalicylic acid colorimetric technique. Phosphatase activity (PME) in soil was analyzed with the p-nitrophenyl phosphate method. The titration method was employed to determine catalase activity (CAT) in the soil [24].

### 2.5. DNA Extraction, PCR Amplification, and Sequencing

Total genomic DNA was extracted from 0.5 g of soil using the FastDNA^®^ SPIN Kit for Soil (MP Biomedicals, Santa Ana, CA, USA), following the manufacturer’s instructions. The integrity of the DNA was assessed using 1% agarose gels, while purity and quantity were evaluated with a NanoDrop™ 2000 and a Qubit fluorometer. Qualified DNA was stored at −20 °C. Bacterial communities were profiled by amplifying the V3–V4 region of the 16S rRNA gene using primers 341F (5′-CCTACGGGNGGCWGCAG-3′) and 806R (5′-GGACTACHVGGGTATCTAAT-3′). Libraries were quantified using Qubit and qPCR, and subsequently sequenced by Novogene (Beijing, China) on an Illumina MiSeq platform. Fungal communities were not profiled by ITS sequencing in the present dataset; therefore, fungal community responses and bacteria–fungi interactions are beyond the scope of this work and are suggested for future studies.

### 2.6. Statistical Analyses

All statistical analyses were conducted using R (version 4.5.1). To mitigate bias arising from variations in sequencing depth, the ASV table was initially rarefied to a uniform depth with the “phyloseq” package. Alpha diversity was computed from the ASV table using the “vegan” package, and intergroup differences were analyzed via one-way ANOVA. When the assumptions of normality (Shapiro–Wilk test) and homogeneity of variance (Levene’s test) were satisfied, Fisher’s LSD post hoc test was employed (two-tailed, α = 0.05). In cases where these assumptions were not met, variables were either log-transformed or analyzed using nonparametric methods. Community composition was aggregated at the phylum and class levels, with the top 10 taxa selected based on average relative abundance, while the remaining taxa were grouped as “Other” for visualization in stacked bar charts. β-diversity was assessed using Bray–Curtis distance, followed by constrained principal coordinate analysis, with significance evaluated through 999 permutation tests and visualized using ggplot2. For community-environment correlation analysis, Pearson correlations were initially calculated for environmental factors, and significance was annotated on the heatmap. Subsequently, Mantel tests were performed to assess the relationship between microbial community distance (Bray–Curtis) and environmental distance (Euclidean) using the “linkET” package. The co-occurrence network retained ASVs that met the specified occurrence frequency threshold. Spearman correlations were computed, and edges were recorded only if they simultaneously met the Bonferroni correction criteria with |R| ≥ 0.85 and *p* < 0.001. To ensure robust treatment-level networks, subnetworks were generated separately for each replicate sample, and edges consistently present across all replicates were selected as the final edge set for that treatment. Network topology comparisons were conducted by calculating and reporting the number of nodes, number of edges, average degree, and counts of positive and negative edges. Networks were exported in GEXF format and optimized using Gephi (version 0.10.1).

## 3. Results

### 3.1. Correlations Between Bacterial Community and Environmental Factors

**Figure 2:**
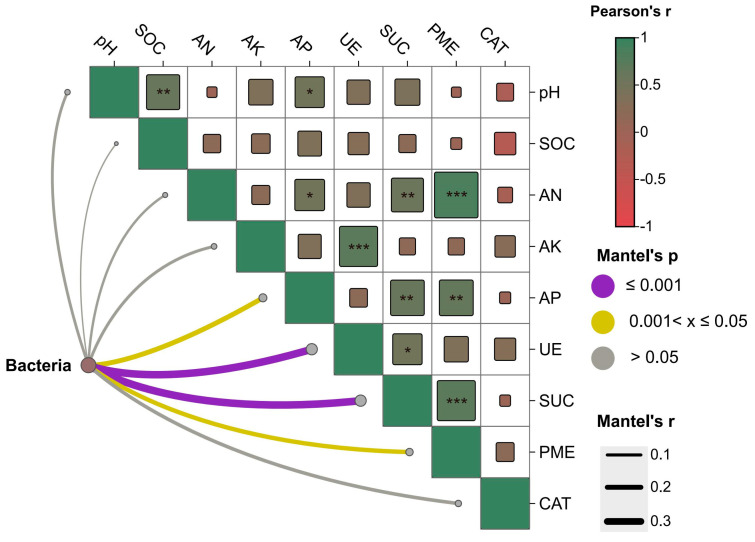
Correlation analysis by Mantel’s test. Note, SOC, organic carbon; AN, available nitrogen; AK, available potassium; AP: available phosphorus; UE, urease activity; SUC, sucrase activity; PME, phosphatase activity; CAT, catalase activity. Correlation analysis and Mantel’s test were performed by Spearman’s method. ***, *p* < 0.001; **, *p* < 0.01; *, *p* < 0.05.

Across all samples, hydrolytic enzyme activities (UE, SUC, PME) were the dominant environmental correlates of bacterial community variation (Mantel, *p* ≤ 0.001) (Figure 2). Available phosphorus (AP) showed a moderate association (0.01 < *p* ≤ 0.05), whereas pH, SOC, AN, AK, and catalase (CAT) were not significant (*p* > 0.05). Pairwise Pearson correlations among edaphic variables were largely positive, with enzyme activities co-varying with nutrient pools (AK/AN/AP) and with each other, while correlations involving SOC or pH were weak or absent (Figure 2). Collectively, these patterns indicate that the proximate gradient structuring bacterial communities aligns with a nutrient-turnover axis defined by C–N–P hydrolysis rather than by single bulk properties.

### 3.2. Soil Bacterial Alpha and Beta Diversities

**Figure 3:**
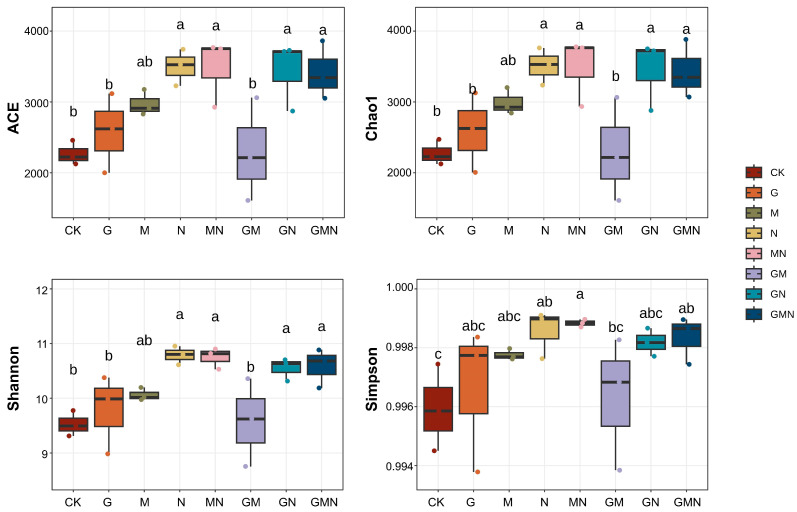
ACE, Chao1, Shannon, and Simpson values of the bacterial communities under different treatments. CK, control; G, RAE; M, *Trichoderma*; N, cattle manure; MN, *Trichoderma* and cattle manure; GM, RAE and *Trichoderma*; GN, RAE and cattle manure; GMN, RAE and *Trichoderma* and cattle manure. Treatments not sharing any lowercase letters are significantly difference (*p* < 0.05, LSD test).

The figure shows the effects of different treatments on soil bacterial α-diversity indices (ACE, Chao1, Shannon, and Simpson) (Figure 3). Compared with CK and G, manure-containing treatments (N, MN, GN, GMN) exhibited significantly higher richness (ACE, Chao1) and diversity (Shannon) (*p* < 0.05). Among fertilized treatments, GM (RAE and *Trichoderma*) showed the lowest richness, approaching control levels. Overall, manure application, either alone or in combination with other amendments, markedly enhanced bacterial richness and diversity, while RAE in combination with *Trichoderma* provided minimal benefits.

**Figure 4:**
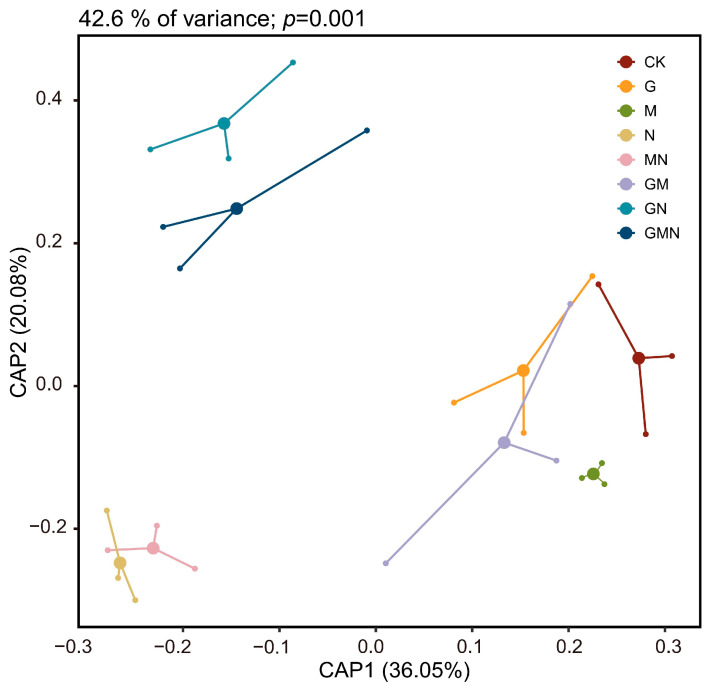
CAP biplots display the differences in bacterial consortia associated with various treatments. CK, control; G, RAE; M, *Trichoderma*; N, cattle manure; MN, *Trichoderma* and cattle manure; GM, RAE and *Trichoderma*; GN, RAE and cattle manure; GMN, RAE and *Trichoderma* and cattle manure.

Constrained analysis of principal coordinates (CAP) (Figure 4) revealed clear treatment-wise separations in community composition (CAP1 = 36.05%, CAP2 = 20.06%; *p* = 0.001). Treatments containing cattle manure (GN, GMN) clustered distinctly from all other groups along CAP2, indicating substantial shifts in bacterial assemblages relative to non-manure treatments. In contrast, RAE–*Trichoderma* (GM) and manure-free treatments (CK, G, M) were positioned closer together, suggesting greater compositional similarity. The manure-only (N) and manure–*Trichoderma* (MN) treatments formed a separate cluster along negative CAP1 values, further highlighting the strong influence of manure application on community structure. Combined with α-diversity results, these findings indicate that cattle manure not only enhanced bacterial richness and diversity but also drove pronounced compositional differentiation, whereas RAE in the absence of manure had minimal effects on community structure.

### 3.3. Bacterial Community Composition at the Phylum and Class Levels

**Figure 5:**
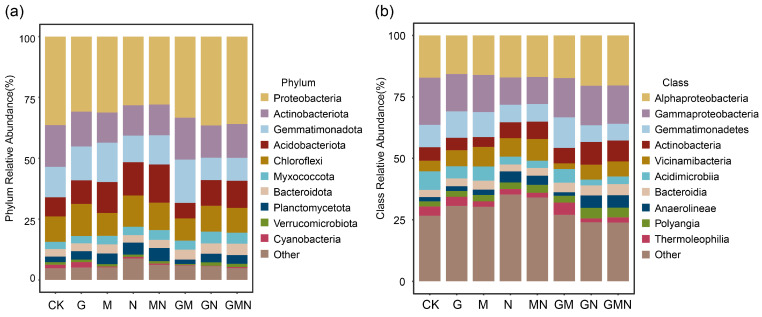
Relative abundance of soil bacterial communities at the phylum (**a**) and class (**b**) ranksunder different treatments. CK, control; G, RAE; M, *Trichoderma*; N, cattle manure; MN, *Trichoderma* and cattle manure; GM, RAE and *Trichoderma*; GN, RAE and cattle manure; GMN, RAE and *Trichoderma* and cattle manure.

At the phylum level (Figure 5a), the dominant bacterial groups across all treatments were Proteobacteria, Actinobacteriota, Gemmatimonadota, Acidobacteriota, and Chloroflexi, together accounting for the majority of the community composition. Compared with CK, Treatments containing cattle manure (N, MN, GN, GMN), generally exhibited a higher relative abundance of Acidobacteriota accompanied by a lower proportion of Gemmatimonadota. In contrast, GM treatment showed a reduced contribution of Acidobacteriota but relatively higher proportions of Gemmatimonadota than the manure-combined treatments.

At the class level (Figure 5b), Alphaproteobacteria, Gammaproteobacteria, Gemmatimonadetes, Actinobacteria, and Vicinamibacteria were predominant. Treatments containing cattle manure (N, MN, GN, GMN) were characterized by enrichment of Actinobacteria and Vicinamibacteria. The GM treatment contained a relatively lower proportion of Vicinamibacteria and a higher proportion of Gemmatimonadetes. In all treatment groups, Alphaproteobacteria and Gammaproteobacteria consistently occupied significant positions, but their relative proportions varied depending on the different combinations of amendments.

### 3.4. Modulation of Microbial Network Structure Under Different Treatments

**Figure 6:**
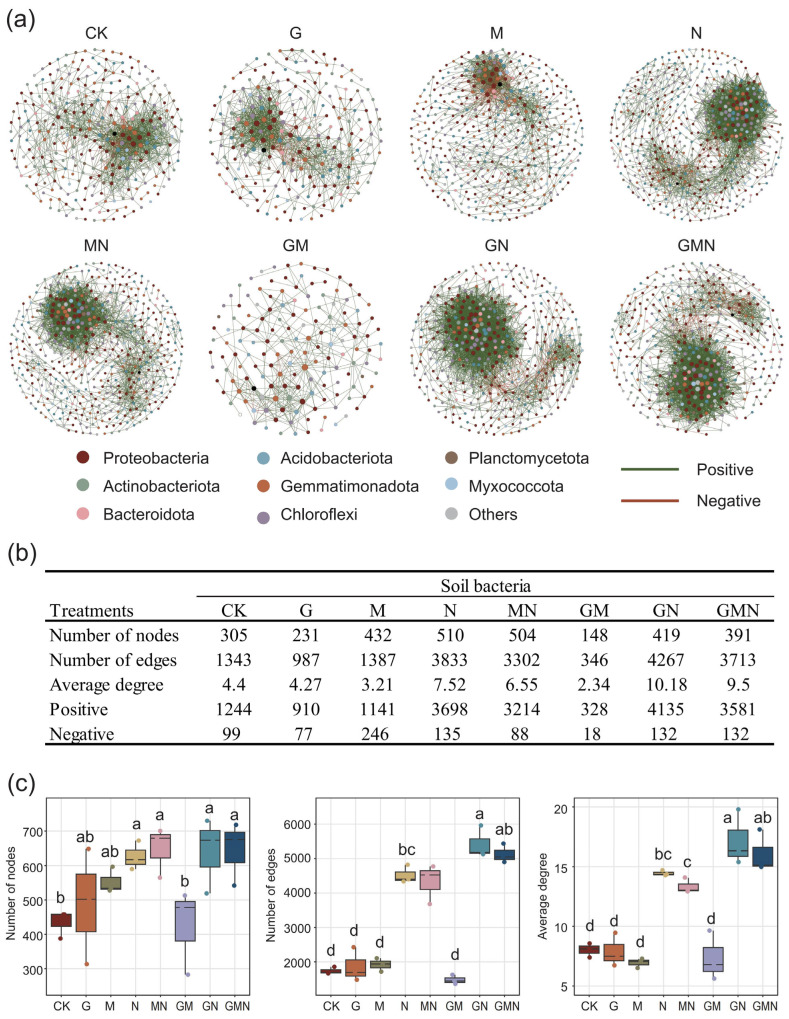
Co-occurrence network analysis of soil bacterial communities under different treatments. (**a**) Network structures at the phylum level, where nodes represent bacterial taxa and edges indicate significant correlations (green: positive; red: negative). (**b**) Topological properties of bacterial networks, including the number of nodes, edges, and average degree. (**c**) Boxplots showing statistical comparisons of network metrics across treatments. RAE treatments (G, GN, GMN) notably enhanced network complexity, with GN and GMN exhibiting the highest numbers of edges and average degrees, indicating stronger microbial connectivity and cooperative interactions. Different letters above boxplots denote significant differences among treatments (*p* < 0.05).

The co-occurrence network analysis demonstrated that treatments involving RAE markedly influenced the structural complexity of soil bacterial communities (Figure 6a). Compared with the control (CK) (231 nodes), the RAE-alone treatment (G) increased the number of nodes to 305 (Figure 6b) and maintained a relatively balanced proportion of positive to negative associations, suggesting that RAE contributed to stabilizing microbial interactions. More strikingly, when RAE was combined with cattle manure and *Trichoderma* (GN and GMN), the networks exhibited the highest complexity, characterized by dense connections and large numbers of edges (4267 and 3713, respectively) (Figure 6b). These treatments also showed the greatest average degrees (10.18 in GN) (Figure 6b), indicating stronger microbial connectivity and potential cooperative interactions within the soil community. By contrast, the GM treatment yielded a simplified network, suggesting that the beneficial effects of RAE were best realized in the presence of manure.

Taken together, these results indicate that manure-derived substrate heterogeneity, especially when combined with RAE (GN and GMN), reinforces cooperative linkages within bacterial communities, whereas pairing RAE with *Trichoderma* in the absence of manure (GM) likely intensifies resource competition or antagonism, leading to network simplification.

## 4. Discussion

### 4.1. Organic Amendments Regulate Microbial α-Diversity Through Resource Availability and Niche Differentiation

The observed increases in α-diversity (Figure 3) under manure-based treatments (N, MN, GN, GMN) suggest that the addition of organic substrates enriched the soil environment with heterogeneous carbon and nutrient pools [25], thereby broadening ecological niches and supporting the coexistence of diverse microbial taxa [26]. Manure is particularly rich in both labile and recalcitrant organic compounds [27], which can be differentially utilized by fast- and slow-growing microorganisms, promoting niche complementarity and enhancing community richness [28]. In contrast, the GM treatment, combining RAE with *Trichoderma*, showed reduced diversity, which may be attributed to selective resource competition [14]. RAE is rich in soluble carbohydrates and organic acids, which could be preferentially consumed by *Trichoderma*, leading to the suppression of bacterial groups that rely on similar carbon sources [29]. Moreover, certain metabolites secreted by *Trichoderma* (e.g., secondary metabolites with antimicrobial activity) might further inhibit sensitive bacterial taxa [30]. RAE alone produced bacterial communities broadly comparable to the control, indicating that its primary value lies in modulating manure-driven responses rather than independently restructuring communities. These findings highlight that the effects of organic amendments on microbial diversity is also influenced by interactions between substrate quality and microbial functional traits [31,32,33].

### 4.2. Resource Quality and Microbial Functional Guilds Drive Shifts in Community Composition

CAP ordination (Figure 4) indicated that community composition segregated primarily along a manure-driven resource-quality axis, consistent with selective enrichment by organic amendments [8]. In manure-containing treatments, Acidobacteriota increased whereas Gemmatimonadota decreased at the phylum level, while Actinobacteria [34] and Vicinamibacteria were enriched at the class level (Figure 5). This pattern suggests that manure input did not simply promote copiotrophic taxa through nutrient enrichment. Instead, it likely increased humified organic matter, enhanced spatial heterogeneity, and strengthened environmental filtering [35]. This enrichment indicates a selective stimulation of functional groups involved in organic matter transformation and efficient resource use, driven by the complex substrates and nutrients supplied by manure.

In contrast, the GM treatment showed lower Acidobacteriota but higher Gemmatimonadota, together with a decline in Vicinamibacteria. This pattern implies that fungal involvement reshaped bacterial assembly. *Trichoderma* may pre-process the inputs, release metabolites, and alter competitive interactions. As a result, the community may shift toward taxa better adapted to fungus-driven carbon flow and changes in substrate spectra, indicating a fungus-channeled carbon flow that disfavors bacterial decomposers [36]. The presence of Gemmatimonadota remained comparatively stable across treatments, suggesting tolerance to edaphic stress and limited sensitivity to short-term changes in carbon quality [37]. These guild transitions align with CAP separations (Figure 4), where GN/GMN clustered away from non-manure treatments, reinforcing the interpretation that substrate heterogeneity—not bulk soil properties—drives community reassembly [28].

With respect to the persistence of the exogenous *Trichoderma*, our bacterial community patterns indicate a strong dependence on resource context. The *Trichoderma*-only treatment (M) clustered close to CK and G in CAP ordination (Figure 4), suggesting a limited long-term imprint on bacterial community structure in this low-fertility reclaimed soil when applied without additional complex substrates. By contrast, co-application with RAE (GM) reduced bacterial richness (Figure 3) and simplified the co-occurrence network (Figure 6a), which is consistent with stronger ecological activity and/or competitive effects of Trichoderma when labile carbon is available.

Mechanistically, manure provides a broad spectrum of substrates, ranging from soluble monomers to structural polymers, thereby expanding metabolic niche space and enabling niche complementarity between fast- and slow-growing strategists [38,39]. This observation aligns with the enzyme-defined turnover axis (UE, SUC, PME) (Figure 2), which best explains community variation [22]. In contrast, GM likely combines the resource monopolization of RAE’s labile acids and carbohydrates by *Trichoderma* with potential antagonism from fungal secondary metabolites, thereby restricting bacterial access to labile carbon and compressing decomposer guilds [40,41].

### 4.3. Microbial Interactions Underpin Changes in Network Complexity and Ecosystem Functioning

Co-occurrence analyses (Figure 6a) showed that manure—particularly when combined with RAE (GN, GMN)—substantially increased network connectivity, evidenced by higher node numbers, more edges, and greater average degree, which is consistent with previous studies [42,43]. The predominance of positive associations (>85%) (Figure 6b) indicates that diverse organic substrates promoted metabolic cross-feeding and cooperation [44], thereby increasing functional redundancy and the potential buffering capacity of the community [45]. In contrast, the GM treatment produced a simplified co-occurrence network. Interaction breadth declined and ecological buffering weakened [46]. Mechanistically, limited substrate diversity and the preferential uptake of labile carbon by *Trichoderma* curtailed complementary bacterial metabolism [39,45]. After explicitly incorporating resource interactions, the structure of resource supply constrains co-feeding and network stability [47].

Linking network patterns to community assembly, manure-derived substrate heterogeneity expanded niche space [48,49] and selected copiotrophic decomposer guilds (e.g., Actinobacteria, Gammaproteobacteria) [50]. This, in turn, reinforced cooperative linkages and enhanced connectivity under GN/GMN. The counter pattern in GM aligns with resource monopolization and/or antagonism by *Trichoderma* [51], suppressing decomposers and restricting metabolic handoffs among bacteria, thereby simplifying the network. Under the manure and RAE treatment, α-diversity is higher, decomposers are enriched, and positive links are denser. These signals point to a resource-mediated mechanism. Amendment quality drives communities toward cooperative, redundancy-rich networks that sustain nutrient turnover and ecosystem functioning [52].

## 5. Conclusions

Manure-derived substrate heterogeneity was the main factor shaping soil bacterial communities by expanding niche space, enhancing α-diversity, and shifting key guilds such as Actinobacteria and Acidobacteriota. Co-application of manure and RAE (GN, GMN) promoted cooperative microbial networks with higher connectivity and functional redundancy, whereas RAE combined with *Trichoderma* without manure (GM) simplified networks through resource competition. Overall, RAE functioned as a key co-driver of microbial cooperation when integrated with manure, rather than as a standalone driver. RAE-based amendments, particularly when integrated with manure, are effective strategies for improving soil microbial functionality in degraded ecosystems.

A limitation of the present work is that microbial community profiling was restricted to bacteria, and therefore we did not characterize fungal community responses or bacteria–fungi interaction networks under the different organic amendment and *Trichoderma* treatments. Given the functional importance of fungi in litter turnover and soil aggregation, future studies should incorporate ITS-based fungal profiling to evaluate how fungal taxa, including *Trichoderma*, interact with bacteria to shape microbiome assembly in reclaimed coal gangue soils.

In addition, direct tracking of the exogenous *Trichoderma* across time would allow estimation of survival dynamics and colonization success, and help link inoculant persistence to the observed shifts in bacterial community structure, enzyme activities, and network properties.

## Data Availability

The original contributions presented in this study are included in the article. Further inquiries can be directed to the corresponding author.
